# Molecular Approaches for the Classification of Microbial Pathogens of Public Health Significance

**DOI:** 10.1155/2014/725801

**Published:** 2014-02-18

**Authors:** Hiroshi Asakura, Holger Brueggemann, Sou-ichi Makino, Yoshiko Sugita-Konishi

**Affiliations:** ^1^Division of Biomedical Food Research, National Institute of Health Sciences, Kamiyoga 1-18-1, Setagaya-ku, Tokyo 158-8501, Japan; ^2^Department of Biomedicine, Aarhus University, Wilhelm Meyers Allé 4, Bartholin Building 1242, Room 433, 8000 Aarhus C, Denmark; ^3^Department of Living Science, Kyoto Seibo College, Fukakusa Taya-cho, Fushimi-ku, Kyoto 612-0878, Japan; ^4^Department of Food and Life Sciences, The Graduate School of Life and Environmental Sciences, Azabu University, 1-17-71, Fuchinobe Chuo-ku, Sagamihara, Kanagawa 252-5201, Japan

A number of pathogenic microorganisms are widely disseminated, surrounding us and sometimes infecting our body, causing a wide range of health problems, ranging from mild to life-threatening diseases. It is now widely accepted to use a variety of molecular approaches to classify or type the causative microbial pathogens in both host and hostile environments, which improve our global understanding on the epidemiology, pathogenesis, ecology, and evolution of the microorganisms, contributing to the prevention of infection, better infection control, and environmental sanitation.

This special issue focuses on molecular approaches for microbial classification and typing, which would be contributable to our better understanding on the microbial features, risks, and potent strategies for its controlling from the viewpoint of public health.

Microbial infections in humans could occur in a number of ways, including food-borne, human-to-human, or environmental transmissions. To reduce the food-borne infections, it is well recognized that the control of microbial safety in foodstuffs is a first-line of preventive strategy. Such an assessment of 126 natural cheese products manufactured in Hokkaido, Japan, was reported by F. K. Esho et al. The authors examined prevalence of some pathogens (*Listeria monocytogenes*, pathogenic *Escherichia coli*, and *Salmonella* spp.) as well as enumeration of indicator bacteria, revealing no detection of those pathogens despite the detection of coliforms in 25 of 126 tested samples (19.8%). Considering the use of pasteurized milk, it could be evidenced that the microbiological quality and hygienic status of the natural cheese tested in this study were in the most fine and satisfactory status.

Microbial quality of meat processing at slaughter is now one of the most public health concerns because of the frequent contamination with a numbers of pathogenic bacteria in meat products attributing to human infections [1]. In this relation, H. Asakura et al. reported the prevalence of Shiga toxin-producing *E. coli* (STEC) O157 in bovine feces and bovine offal at preslaughter and their characterization. At preslaughter, the STEC O157 was detected in 31 of 301 cattle feces (10.3%). Throughout slaughtering, this pathogen was detected from bovine offal and carcasses, and some of which exhibited identical macrogenotypes, suggesting their cross-contamination at preslaughter.

Development of the protocol for the detection of foodborne pathogens was reported by Hayashi et al. The authors demonstrated a quick screening methodology by the development of cocktail PCR dipstick DNA chromatography (CPDC) assay, which enabled finalizing the simultaneous detection of multiple enteric pathogens including *Salmonella* spp., *Shigella* spp. enteroinvasive *E. coli* (EIEC), and enterohemorrhagic *E. coli* (EHEC) from food samples, within 45 min after 4–6 h of enrichment in a recently developed FPE broth [2]. Such method offers rapid report to food suppliers and helps the quick shipment of safety-confirmed food products to markets.

*Vibrio cholerae* is an aquatic bacteria that causes cholera, a major public health problem especially in developing countries [3]. For the rapid and sensitive detection of this pathogen, E. Yamasaki et al. report the development of immunochromatographic test strip targeting cholera toxin (CT-IC). The authors evaluated the high sensitivity (detection limit of 10 ng/mL) and no cross-reactions of this developed tool, confirming its feasible use for the rapid detection and surveillance of toxigenic *V. cholerae* that is a public health threat. The rapid detections in early stage of epidemic would also allow quick triggering of control measures.

Some pathogens could withstand under hostile environments. *Legionella pneumophila* that causes Legionnaires' disease and Pontiac fever [4] is one of the representatives to achieve adaptation to aquatic environments. The article of M. Tachibana et al. reports the prevalence and virulence characteristics of this pathogen in environmental water and foot spa in Yamaguchi, Japan. Finally, *L. pneumophila* was isolated from 5 of 22 samples, which exhibited virulence characteristics to humans. The authors thus concluded the potent risks for the transmission of this pathogen from the spa via generated aerosols.

*Staphylococcus aureus*, a gram positive coccal bacterium, is either commensal that colonizes healthy nasal mucosa or pathogen of humans [5]. During the last five decades, *S. aureus* clones that resist methicillin (methicillin-resistant *S. aureus*, MRSA) disseminated and caused a medical and public health problem worldwide [6]. N. Indrawattana et al. performed genotypic and phenotypic classification of 92 *S. aureus* isolates from periodic monitoring in Thailand. The authors confirmed the link between the possession of virulence genes and resistance to methicillin as well as the fact that about 73% of the isolates formed biofilms on abiotic surface. The results of this study provide insight information on molecular and phenotypic markers of *S. aureus* clinical isolates in Thailand which should be useful for future active surveillance that aimed to control a spread of existing antimicrobial resistant bacteria as well as early recognition of a newly emerged variant.

In summary, this special issue covers a range of diverse topics related to the microbial classification of public health significance. We hope the papers published will serve to further highlight the microbial safety in foods and environments, as well as in stimulating further researches into the virulence features of microbes and development of diagnostic tools, thereby contributing to the improved patient treatment and microbial safety in sources of infection.
